# Conventional Thermoset Composites and Their Sustainable Alternatives with Vitrimer Matrix—Waste Management/Recycling Options with Focus on Carbon Fiber Reinforced Epoxy Resin Composites

**DOI:** 10.3390/ma18020351

**Published:** 2025-01-14

**Authors:** Paraskevi Markouti, Evanthia Tzouma, Alkiviadis S. Paipetis, Nektaria-Marianthi Barkoula

**Affiliations:** Department of Materials Science and Eng, University of Ioannina, 45110 Ioannina, Greece; euamark8@gmail.com (P.M.); e.tzouma@uoi.gr (E.T.); paipetis@uoi.gr (A.S.P.)

**Keywords:** thermoset composites, covalent adaptable networks, vitrimers, recycling, CFRPs, epoxy resin

## Abstract

Carbon-fiber-reinforced polymers (CFRPs) with epoxy matrices are widely applied in high-performance structural applications and represent one of the biggest classes of materials with urgent need for end-of-life management. Available waste management methodologies for conventional thermoset composites with a focus on CFRPs are briefly reviewed and their limitations are highlighted. In the quest to obtain materials with mechanical performance, thermal stability, and sustainability, the research community has turned its interest to develop polymer composites with adaptable and dynamic networks in their matrix, and lately also at an interface/interphase level. The current review focuses on the life extension/waste management options that are opened through the introduction of covalent adaptable networks in the epoxy matrix of CFRPs. The processing conditions that are applied for the healing/repairing, welding/reshaping, and/or recycling of CFRPs are presented in detail, and compared based on the most common dynamic exchange reactions.

## 1. Introduction

Polymers can be divided into two big categories according to their reaction to heat, thermosets, and thermoplastics. Thermoplastics allow their chains to move and flow when heated above critical temperatures (glass transition temperature (Τ_g_) and melting temperature (T_m_), respectively) and, therefore, they can undergo multiple heating and cooling cycles [1]. On the contrary, after curing, the existence of covalent crosslinks restricts the mobility of the macromolecular chains in thermosets; therefore, this class of polymers does not melt or soften during heating cycles [1]. Thus, thermosets are rigid, and insoluble with high tensile strength, stiffness, fragility, and long-lasting endurance, while the lack of permanent covalent connections in thermoplastics allows them to be easily processed and recycled. Another important class of polymeric resins is elastomers. While most elastomers are crosslinked, thermoplastic elastomers do exist, and they can be manufactured following conventional techniques applied to thermoplastics. For structural applications, polymers are being reinforced with high-performance fibers [2]. Fiber-reinforced polymer composites could be either thermoset-, thermoplastic-, or elastomeric-matrix. Strength, stiffness, better creep behavior, fiber wetting, and improved adhesion are some significant characteristics that bring thermoset matrices to an advantageous position over thermoplastic resins in high-performance applications [3]. Easier processing and low-temperature manufacturing options are also advantageous characteristics of the thermoset matrices; however, the chemical reaction of cross-linking is slow and demands heat input for its acceleration, while toxic gases could be potentially released during manufacturing [4]. Finally, an elastomeric matrix is usually preferred in applications that demand high toughness and strain at break. However, due to the T_g_ of elastomers that is considerably below room temperature, their composites are not conventionally used in applications that require high stiffness/strength at higher temperatures. The most important drawback of thermoset-based composites is that their matrix cannot be remolded or reshaped limiting their repairing, reuse, and recycling capabilities [3,4,5,6]. Thermoset composites are commonly disposed of in landfills or incinerated after the end of their useful life, but these methodologies are not preferred due to their environmental impact [7]. There is an ongoing exploration to evolve recycling into a sustainable solution for managing end-of-life thermoset composites [7,8]. Mechanical recycling is the most commercially applicable technique but results in the deterioration of performance, while chemical and thermal recycling are more efficient methods for obtaining high-end products [8,9,10].

Since the matrix is introducing restrictions for the sustainable use and disposal of thermoset composites, efforts are being made to alter the three-dimensional network of thermoset systems and make them reprocessable, repairable, and/or recyclable [11,12,13,14,15,16]. It has been suggested that all these features can be exploited in cross-linked polymer networks through the effective exchange of chemical cross-links between the organic polymer chains. Covalent adaptable networks or CANs are polymer networks that contain exchangeable bonds and can achieve macroscopic flow. Depending on the exchange mechanism that prevails they are termed as dissociative and associative [17,18]. In the first type of exchange, the chemical bonds are initially disrupted and then formed again, whereas, in the second type, the disruption of the original cross-linkage occurs only after a new covalent bond has already been formed. Leibler and coworkers in 2011 designed epoxy networks with associative exchange reactions, that presented Arrhenius-like gradual viscosity variations, and for this reason, they named this class of materials vitrimers [19]. While it has been suggested that CANs with dissociative exchange mechanisms exhibit a more rapid decrease in viscosity with increasing temperature, it has been recently argued that dissociative chemistries also show an Arrhenius response under typical reprocessing conditions, indicating that both types of CANs behave almost identically over broad temperature ranges [18]. The development of such adaptable polymer networks leads the way for new waste management strategies that will address the challenges of environmental sustainability of high-performance thermoset composites [20].

The aim of this brief review is twofold: (a) to provide a brief overview of the different recycling methodologies of thermoset composites, with a focus on carbon-fiber-reinforced composites (CFRPs) with epoxy matrices, which is the biggest representative of high-performance polymer composites, and (b) to discuss in detail the possibilities that are opened in terms of waste management of high-performance thermoset composites through the introduction of more sustainable matrices, like epoxy-based vitrimers. Thus, the current manuscript is organized as follows: in § 2, a brief overview of the main waste management options of CFRPs with conventional epoxy matrices (i.e., mechanical, thermal, and chemical recycling) is presented, while § 3 provides a thorough review of the recently published works on CFRPs with epoxy vitrimer matrices. The healing/repairing, welding/reshaping, and/or recycling options of the dynamic CFRPs are grouped based on the exchange chemistries of the vitrimer matrix (i.e., ester-, imine-, disulfide-, and other exchange bond reactions). Finally, a direct comparison of the processing conditions during life extension and/or end-of-life management of the different dynamic CFRPs is presented for the first time in this review article.

## 2. Overview of Recycling of Thermoset Composites with Focus on CFRPs with Epoxy Matrices

Since the critical assessment of recycling of thermoset composites by Pickering [21] in 2006, a lot of progress has been made around the recycling of such composites, with an emphasis on CFRPs, which has been discussed in recent review articles [7,8,9,10,15,20,22,23,24,25,26,27,28,29,30,31,32,33,34,35,36,37,38,39,40,41,42]. The focus on recycling of CFRPs is due to their increasing use in aerospace, civil engineering, and other fields which results in a high volume of waste. Epoxy resins, which have been commercially available since the 1950s, are one of the main matrices in CFRPs nowadays. The epoxy composites’ production is expected to grow to 4 Mt in 2030 [8]; however, it is recognized that difficulties in the repairing, reshaping, dismantling, and recycling of epoxy composites are obstacles when they approach their end of working life. Most of the research findings around the recycling of epoxy-based CFPRs were obtained during previous decades, and, as aforementioned, are already extensively reviewed. The next paragraphs (i.e., § 2.1–2.3) give an overview of the respective steps/conditions that are applied under each recycling methodology, provide a summary of the main research outcomes, and try to highlight new findings, published since 2020, towards more efficient, and financially viable solutions with a lower environmental impact.

### 2.1. Mechanical Recycling

**Figure 1:**
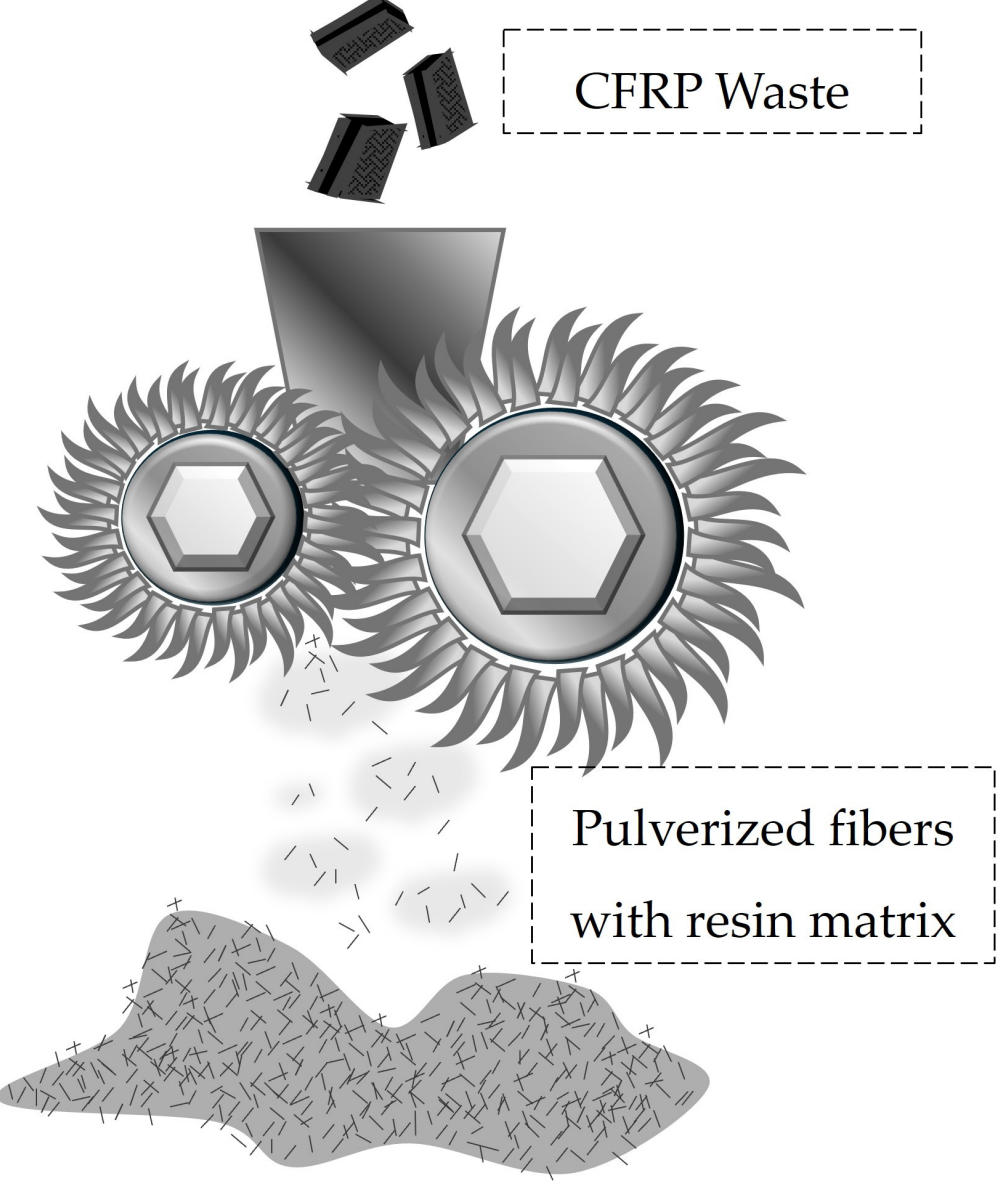
Schematic representation of the grinding process of CFRP waste, and the resultant flakes containing pulverized fibers with resin.

Mechanical recycling often starts with the reduction in the size of the thermoset composites, as schematically illustrated in Figure 1, into 50–100 mm flakes, by using a slow-speed process, followed by high-speed cutting into sizes ranging between 10 mm and 50 μm [5,10,21,22].

Recent studies have shown that elevated cutting speeds can lead to the loss of the coating of the cutting tool, resulting in a lower amount of recycled CFRP particles. While the cutting speed is connected to the process efficiency, it does not affect the quality of the recycled particles, since, as demonstrated, the mechanical and thermal properties of the resulting compounds are not significantly altered [43]. The crushing process operates at room temperature and atmospheric pressure and the processing time ranges mostly from 40 min to several hours [9,44]. Depending on their size, they are mostly used in the building and construction industry, as fillers/short fibers in bulk molding and sheet molding compounds for automotive and electrical parts, and as reinforcement in thermoplastic matrices [10,45,46,47,48,49,50]. Another proposed application of recycled CFRPs is filler in antistatic coatings and electrically conductive plastics [40]. Overall, it has been concluded that mechanical recycling is a cost-effective, simple technique with low environmental impact. However, the size reduction substantially decreases the value of high-performance fibers, like carbon ones, used in structural thermoset composites, and the recycled fibers/fillers, cannot compete with conventional reinforcement on an economical and performance scale [23,27]. Therefore, the research interest in recycling high-performance thermoset composites has been concentrated on thermal and chemical methodologies.

### 2.2. Thermal Recycling

**Figure 2:**
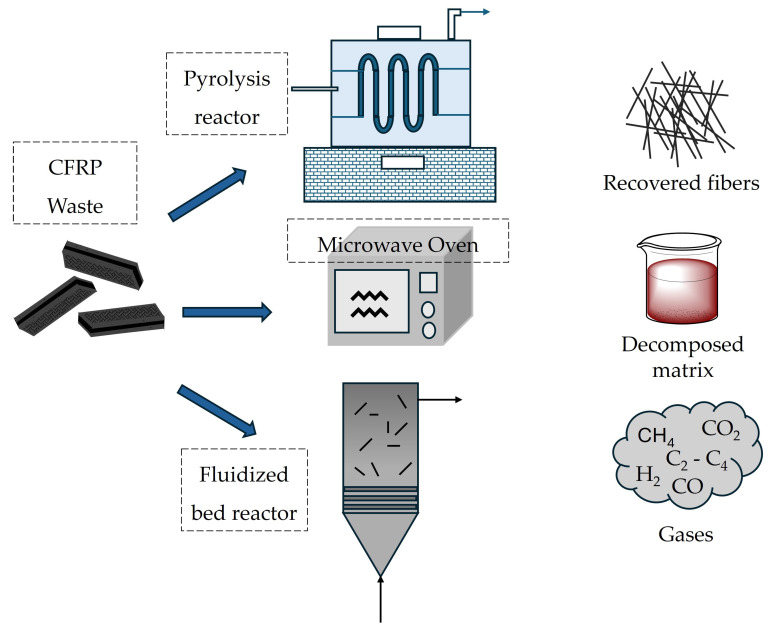
Schematic representation of the thermal recycling process of CFRP waste, the resultant fibers, and decomposed liquid/gaseous matrix products.

Thermal recycling processes demand high-temperature treatment. They can be categorized into pyrolysis (conventional and microwave-assisted) and fluidized-bed techniques, as schematically illustrated in Figure 2.

Conventional pyrolysis is conducted in most cases at 400–1000 °C, at atmospheric pressure, over 15 min to several hours. During pyrolysis, the matrix is decomposed into gases or liquids, which can be used as sources for fuels and chemicals, while the recovery of the fibers is enabled [31]. The advantage of pyrolysis in the case of CFRPs is that it results in carbon fibers that maintain, to a large extent, their length, strength, and other functional properties, like electrical conductivity [32,33,34]. At the same time, it is recognized that the reaction conditions need to be closely controlled to minimize the carbon residues [24,32,33,34], while the process cannot be characterized as very environmentally friendly due to the toxicity of the emitted gases [24]. A new method for recycling CFRP composites by low-temperature pyrolysis in combination with pre-treatment by solvolysis showed that the heat required after pre-treatment for fiber recycling was reduced and there was an improvement in the mechanical properties of the fibers (approximately 10% higher tensile strength than that of the conventional method) [51]. Quite recently, it has been suggested to assist the pyrolysis process by the application of microwave technology [9,24,25,26,34]. Compared to the conventional pyrolysis method, microwaves allow the selective heating of the fibers and result in the reduction in the required amount of energy (approximately 10 vs. 21 MJ/kg), time, and temperature [34,52], since 20–30% lower energy losses have been documented over conventional conduction/convection heating [53]. In addition, the recovered fibers are cleaner and the whole process is more efficient, more environmentally sustainable, and less costly [34,52,54,55]. Carbon dioxide (CO_2_) emissions from microwave pyrolysis were 544 t CO_2_ eq. compared to 744 t CO_2_ eq. for conventional pyrolysis and the total cost of the microwave process was EUR 5.60/kg compared to EUR 12.00/kg for the conventional process [52]. However, the scale-up of the technique at an industrial level has not been yet demonstrated [34].

The fluidized-bed technique involves the use of a fluidized bed of silica sand, where the composite waste is placed in a crushed form (less than 25 mm in size), and the separation is carried out in a range of temperature (e.g., 400–650 °C) and pressure (10–25 kPa) conditions using airflow over several hours leading to CO_2_ emissions [31,52]. The matrix material decomposes allowing to reclaim the fibers without any resin excess [36], but the resin cannot be recovered, as in the pyrolysis process [21,37]. Oxidation of the organic by-products at higher temperatures can however enable the reuse of the decomposed matrix [10,38]. In relation to other comparable procedures, fluidized bed recycling is more efficient in recovering fibers (approximately 90%) and less energy-intensive (approximately 9 MJ/kg). However, it is costly due to the maintenance of a continuous flow of hot air (approximately EUR14/kg) and is not environmentally friendly due to the emission of pollutants (organic solvents and gases (615 t CO_2_ eq.)). Furthermore, the length of the fibers is shorter compared with the one obtained after the conventional pyrolysis, the mechanical properties are reduced (75% vs. 78%), and, as aforementioned, the recovery of the polymer matrix is limited [31,52,55].

Finally, combustion/incineration is a simple thermal technique that can be applied at an industrial level for the waste management of thermoset composites. Although this technique mostly results in energy and not in material recovery, in specific cases, e.g., co-processing of thermoset composite waste in cement production, apart from the savings in energy, it is possible to use the incombustible residues and fibers as raw material for the cement clinkers [8]. Incineration can process mixed waste, but it is the least preferable for the waste management of thermosets and their composites because of the pollutants that are released through this process [39].

### 2.3. Chemical Recycling

**Figure 3:**
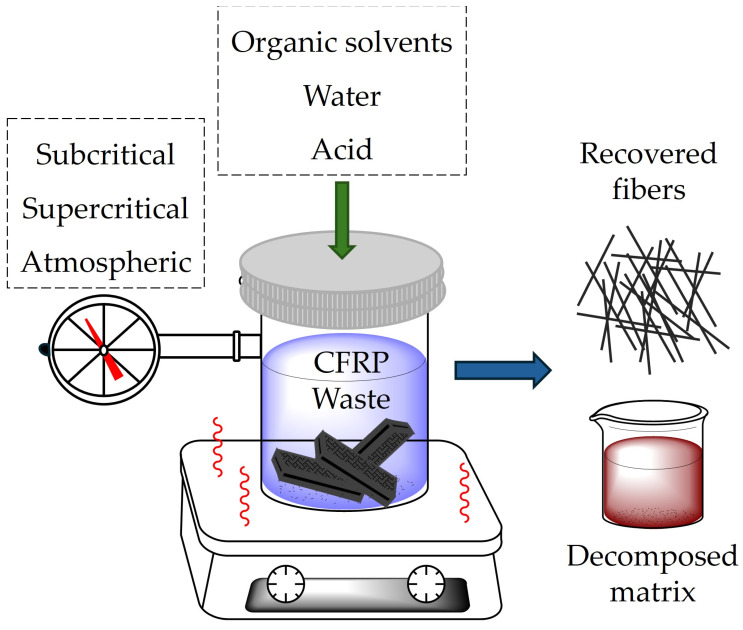
Schematic representation of the chemical recycling process of CFRP waste, and the resultant fibers and decomposed matrix products.

Chemical recycling aims to remove the matrix and release the fibers through the chemical decomposition of the thermoset resin under mild conditions or using super- and subcritical fluids, with or without the presence of catalysts [40,41,42], as schematically illustrated in Figure 3.

The matrix material can be re-polymerized or used as fuel [7]. Solvent-based methods, including super- and sub-critical fluids, seem to be the most effective ones for the recycling of the epoxy matrix of CFRPs since they target the decomposition of the ester, C–N, and C–O bond of the epoxy resin [56]. An overview of some indicative recent publications (since 2020) on the chemical recycling of CFRPs is provided in Table 1. The dissolution of the matrix is conventionally enabled by water, organic solvents, and/or acid and results in the recovery of the fibers that retain their mechanical properties [10,40,41,42]. Supercritical and subcritical states facilitate the efficient decomposition of the matrix in CFRP composites due to the high diffusivity and mass transfer of the fluids under these states [7,41,42]. Recycling with super/subcritical fluids applies temperatures in the range of 250 °C to 450 °C and high pressures (e.g., 5–35 MPa) [9]. Thus, to achieve the required critical points, temperature and pressure need to be regulated and this results in increased energy costs and potential deterioration of the performance of the reclaimed fibers [10,42].

In the case of chemical solvents, normally atmospheric pressure is applied and the temperature ranges between 90 °C and 350 °C. Recent studies on the chemical recycling of CFRPs under mild conditions have revealed that mixing oxidizers with acids and ketones is an efficient way to degrade thermoset composites in less time and by using less heat [10,41,42]. As observed in Table 1, the combination of solvents with catalysts has also been shown to result in relatively quick degradation under mild conditions (temperature <200 °C).

Furthermore, it has been demonstrated that chemical recycling allows the reclaim of high-performance carbon fibers (CF) with comparable properties to virgin ones, with the potential to reuse the recovered matrix. Therefore, the process can be characterized as a sustainable route due to its very high recovery efficiency in solvents and recycled products and use, in most cases, of environmentally friendly chemicals and mild reaction conditions [41,42]. However, highly concentrated acids and bases may raise concerns due to their oxidative and corrosive behavior along with their toxicity, lowering the environmental friendliness of these routes [9]. Electrochemical decomposition has also been recently used for the recycling of CFRPs [40,41]. While easy to apply and not very demanding in terms of equipment cost, this method is time-consuming and may result in the deterioration of the mechanical properties of the CF if the applied voltage is not well controlled [40,41]. To summarize, three types of techniques have been explored for the end-of-life management of CFRPs: the chemical, thermal, and mechanical ones [6,7,8]. Thermal methods normally result in energy recovery, chemical methods aim to recover the building blocks of the composites, while mechanical methods are mainly based on the reduction in the size and reuse of the material without the separation of the different constituents. The simultaneous treatment of both matrix and fiber, without the need for separation of the constituents of the composites, can happen through the application of mechanical methods (pulverization) or thermal methods (combustion, fluidized bed, and pyrolysis). The removal of the matrix and subsequent recovery of the fiber can be better supported by chemical recycling methodologies. The discussed processes use distinct processing conditions and have different environmental impacts. Therefore, a direct ranking of the environmental and cost efficiency of the different recycling processes is not possible, and real-case scenarios need to be assessed with life-cycle analysis methodologies for comparison purposes [62,63,64,65].

## 3. Waste Management of Vitrimer Composites with Focus on Epoxy-Based Systems

### 3.1. Waste Management Options Enabled by the Introduction of CANs

**Figure 4:**
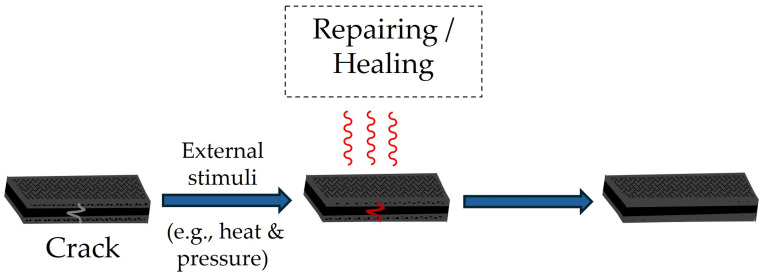
Schematic representation of the repairing/healing process of vitrimer composites, activated through the application of external stimuli.

As discussed in the introduction, CANs can rearrange their network through externally activated reactions enabling the repair of discontinuations (e.g., defects, damage, etc.) that are created during their manufacturing, assembly, and/or service [13,16,17,66], as schematically illustrated in Figure 4. This is of paramount importance, when such materials are applied as a matrix in thermoset composites, due to the potential for a substantial extension of the service life, minimization of maintenance costs, and reduction in composite wastes [11,13,14].

**Figure 5:**
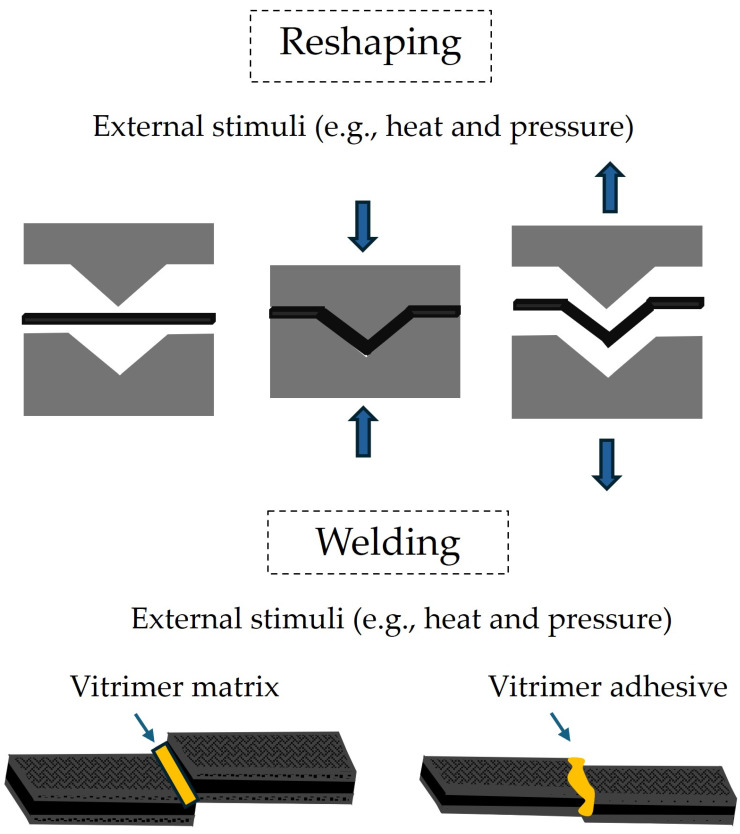
Schematic representation of the reshaping/welding processes of vitrimer composites, activated through the application of external stimuli.

Another very important aspect that can have a tremendous impact in managing end-of-life composites is the ability of CANs to alter their shape and/or be repeatedly welded due to their topology change that can be activated by thermal, photochemical, and other stimuli (e.g., water, organic solvent, pH change, etc.) after curing [5,8,14,17,67]. This enables CAN-based composites to be re-manufactured/re-shaped/re-bonded and repositioned in the market and facilitates the dismantling of composites from various structures which supports their recycling possibilities [14,17], as schematically illustrated in Figure 5.

Depending on the type of exchange (associative or dissociative) different processing techniques may be applied for the remanufacturing/reprocessing/mechanical recycling of CAN composites. This is mainly linked to the time frame for stress relaxation and the viscosity drop upon activation, which is more pronounced in the case of dissociative exchange chemistry. Thus, dissociative chemistry allows the use of processing techniques in the liquid state (e.g., injection and/or extrusion molding) while associative exchange chemistry demands the application of external stress to facilitate the flow of the matrix within seconds and fits better with compression molding processing methodologies [5].

**Figure 6:**
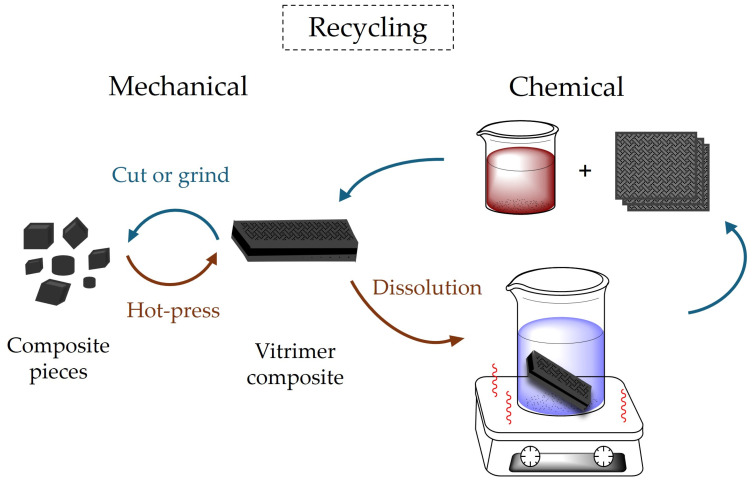
Schematic representation of the mechanical and chemical recycling processes of vitrimer composites.

In terms of recycling, mechanical and chemical are the two main routes that have been explored in CAN composites, as schematically illustrated in Figure 6. Contrary to conventional thermoset composites, which mostly use the waste CFRPs after grinding as filler in less demanding applications, mechanical recycling of vitrimer-based CFRPs could result in the development of new composite systems, starting from short-fiber granules that contain both the matrix and the reinforcement. This is mainly supported by the possibility of the matrix to flow and reestablish the polymer network, through the activation of the dynamic exchangeable bonds, under the application of heat and pressure and/or other stimuli. It is also possible to thermally activate the fiber/matrix separation, but this is better supported by the dissociative chemistry compared to the associative one [5]. Further discussion on the recycling possibilities of CFRPs, with epoxy vitrimer matrices will be the subject of the next paragraph.

### 3.2. Epoxy-Based Vitrimer CFRPs—State-of-the-Art Results on Their Healing, Reprocessing, and/or Recycling Studies

**Figure 7:**
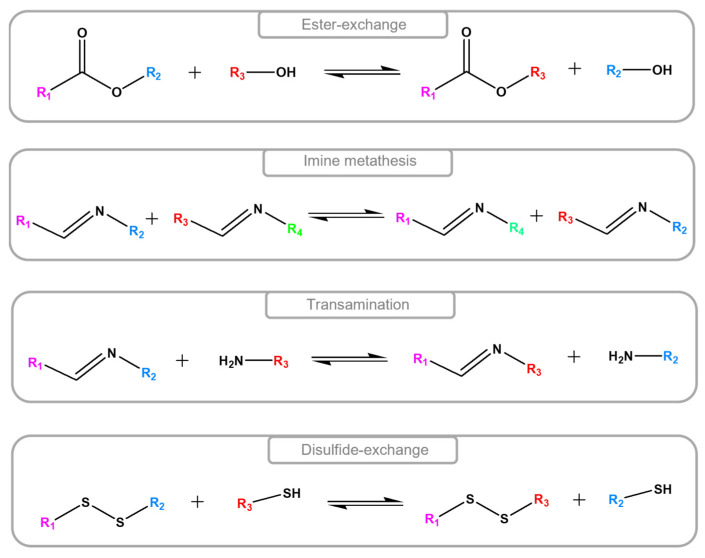
Schematic representation of characteristic dynamic exchange reactions based on ester-, imine-, and disulfide-bonds after [68,69]. Different colors represent different reactive groups.

The number of articles that can be found with the keywords “vitrimer” and “composites” in their title, abstract, and keywords section is over 340 in “Scopus”, while the same number reduces to just over 200 when the keyword “epoxy” is added in the search. Epoxy vitrimers can be synthesized through the introduction of various chemistries, but the ones that have been mainly discussed are the ester-, imine-, disulfide-, and bond exchange reactions [14], which are illustrated in Figure 7. Moreover, bio-based epoxy vitrimers have been in the spotlight for the last decade to further support the development of sustainable thermoset composites [14]. The paragraphs below provide an overview of recent research articles (since 2020) that use vitrimer epoxies as a matrix to fabricate CFRP composites. They include information regarding data relevant to waste minimization/management (i.e., life extension and/or end-of-life management) of the CFRPs. The studies are grouped based on the type of dynamic exchange reaction of the vitrimer matrix that enables the waste minimization/management features of the respective CFRPs. Note that results on the healing/repairing, welding/reshaping, and/or recycling of the epoxy vitrimer are included in the analysis only when respective information is not available for the CFRP composites.

#### 3.2.1. Ester-Exchange Reactions

One of the most popular chemistries for manufacturing epoxy vitrimers is based on the process of exchanging the organic group of an ester with free alcohol [11,14]. Thus, Table 2 includes data from the studies that are based on ester-exchange chemistry. As observed in Table 2, a variety of chemistries have been employed to facilitate ester-exchange reactions in epoxy matrices and enable the recycling of CFRPs. In [70], it was shown that, with an internally catalyzed system, it was possible to obtain shape-changing capabilities with a reprocessing time of approximately 1 h at 200 °C. Furthermore, 100% degradation of the matrix was demonstrated in water at 180 °C in just 5 h due to the hydrolysis of the ester bonds. The hydrolyzed matrix contained oligomers with hydroxyl and carboxyl reactive groups that present the potential for re-polymerization. This, along with the fact that the reclaimed fibers exhibited almost identical properties with the virgin material, supports the potential of the proposed chemistry in terms of chemical recycling. However, it was also shown that the T_g_ of the proposed matrix was up to 95 °C, preventing the use of this chemistry in high-temperature applications. An extensive study on the mode I interlaminar properties of self-healable CFRPs with various interfaces was conducted by Zhao et al. [71]. The mechanical properties of the vitrimer CFRPs were comparable with commercial thermosetting composites, while the T_g_ of the prepared matrices ranged between approximately 50 and 90 °C [71]. The important outcome of the study was that specimens with complete delamination failure could be healed twice under the stimuli of heat and pressure, while the healing efficiency of the fracture toughness was estimated at >80% [71]. The same vitrimer-matrix chemistry was combined with a polylactic acid (PLA)-based microchannel with epoxy resin with the aim of developing CFRP composites with internal and external healing capabilities [72]. This combination resulted in 134.5% healing efficiency; however, the developed matrices showed inferior T_g_ values (below 64 °C) [72]. To enhance the toughness of the matrix material, a polyether-modified epoxy vitrimer with ester-based exchange reactions was developed in [73]. Self-healing, reshaping, and recycling abilities were confirmed for the epoxy vitrimer; however, these features were not examined on the CFRP composite, which was only dissolved in EG to reclaim the CFs [73]. Poutrel et al. [74] proposed a heterogeneous reactive system to manufacture CFRPs with processing properties relevant to industrial applications. The CFRPs showed healing, adhesion, and chemical decomposition abilities; however, the T_g_ of approximately 100 °C and the softening observed in the mechanical performance close to the T_g_ suggests that these systems cannot be effectively used in high-end applications. CFRP vitrimer composites with slightly higher T_g_ (approximately 110 °C) were prepared in [75,76], with a focus on the fracture behavior and reparability of the delamination in CFRPs, which was not, however, considered successful. Zhang et al. [77] developed a series of catalyst-free vitrimer epoxies, with relatively high T_g_ (140–165 °C) and good mechanical performance. They demonstrated the ability of the matrix material to activate topology changes and present self-healing, reshaping, and reprocessing capability, while in the case of the composite, they used the matrix as a reversible glue for welding and reclaimed the CF through the dissolution of the matrix in ethylene glycol (EG). A very promising study was conducted by Hao et al. [78]. The authors not only managed to prepare a vitrimer epoxy with competitive mechanical performance and high T_g_ (approximately 200 °C) but also demonstrated the possibility of fully degrading the matrix of the CFRPs in 5 h at 200 °C and reclaiming almost uninfluenced CF. More importantly, it was shown that it was possible to epoxidize the degradation products of this process and mix them with fresh epoxy to prepare a new epoxy-based vitrimer matrix with reusability and recyclability [78]. The effect of oxidative aging on the dissolution capability of the vitrimer matrix and the purity of the resultant CF in CFRPs with ester bonds was discussed by Fang et al. [79]. It was shown that the aging temperature may result in matrix residues on the reclaimed CF. Furthermore, while aging may facilitate the ester-exchange reactions in the pure matrix, when it comes to the CFRP, it was shown to result in a reduction in the recycling efficiency, which, as suggested, requires further investigation. Barnett et al. [80] focused on the manufacturing of vitrimer organo-sheets using recycled CF and epoxy vitrimer matrix in the form of films. These sheets were then consolidated in laminates, and the healing capability of the composites was confirmed. However, even if more sustainable, it was accepted, that their thermal and mechanical performance falls behind the needs of the aerospace and automotive industry [80].

With the focus on creating fire-safe vitrimer composites, vitrimer matrices with intrinsically flame-retardant properties were developed and tested in [81,82,83,84,85]. Apart from the superior fire resistance, all studies confirmed the possibility of dissolving the vitrimer matrix of the CFRP and reclaiming almost intact CFs [81,82,83,84,85]. Furthermore, it was shown that it was possible to reuse the CF to develop CFPRs with identical flexural performance as the ones with virgin fibers [81]. The possibility of healing internal cracks was also demonstrated on CFRP specimens exposed to an interlaminar shear test, where the healing efficiency was calculated at 93% [82,83]. Apart from reclaiming the CF, the possibility of reusing the matrix degradation products as adhesives and as monomers/oligomers to regenerate the vitrimer matrix was demonstrated in [84,85], respectively.

Bio-based vitrimer matrices were proposed in [86,87,88,89,90,91] to manufacture sustainable CFRPs. The possibility of reprocessing, shape-changing, and welding vitrimer CFRPs was discussed in [86]. Chemical degradation of the epoxy vitrimers and CFRPs was possible at 100 °C after only 30 min by using ethanolamine, and the reclaimed CFs were almost unaffected [86]. It was also observed that the degradation products contained many hydroxyl groups, which makes them reusable as chain extenders and cross-linking agents for bio-based polyurethanes [86]. The ability of bio-based vitrimer CFRPs to activate rearrangements that facilitate repairing, reprocessing, reshaping, welding, and matrix degradation with intact CF has been presented in [87]. Although the feasibility of waste minimization/management has been demonstrated, the mechanical performance and T_g_ (up to 48 °C), cannot qualify the proposed systems for high-performance applications. In the same direction, Li et al. [88,89] prepared vitrimer CFRPs with epoxy–acid/anhydride matrices and a bio-based curing agent. While the study demonstrated quite good mechanical and thermal properties and repairing/reprocessing/recycling options for the matrix material, the data on the CFRP are only limited to the characterization of the reclaimed CF, which shows a similar chemical profile and mechanical performance with the original fibers [88,89]. In addition, the reusability of the degradation products of the vitrimer matrix as chain extenders and crosslinking agents for bio-based polyurethanes was also proposed in [89], however, without further investigation of this option. Continuous efforts have resulted in bio-based vitrimer matrices and respective CFRPs with higher T_g_ (approximately 77 °C [90] and 90 °C [91]) and the possibility to reclaim undamaged CF after the immersion of the CFRPs in a NaOH–ethanol solution at room temperature (RT) [90] or in water at 160 °C [91] in just 2 h. Even with those efforts, such systems cannot yet compete with the conventional or even the petrochemical-based vitrimer-CFRPs for high-temperature applications.

#### 3.2.2. Imine-Exchange Reactions

Another interesting chemistry for CANs is the one based on imine bonds (or else Schiff base) that can be activated through the application of a variety of stimuli (e.g., temperature, solvent, pH, etc.) and can result in both associative (transamination and imine metathesis) and dissociative reactions (hydrolysis) [11,12,14]. Table 3 provides an overview of the studies on vitrimer CFRPs with imine-exchange reactions in their epoxy matrices. As observed, most of the recent studies focused on proposing bio-based matrix components that combine waste management abilities through the activation of imine-exchange reactions with high mechanical performance and/or thermal stability/fire resistance. Then, only studies that introduce imine-exchange reactions in purely petrochemical-based matrix systems use matrix chemistries that combine a conventional epoxy resin with polyetheramine and terephthalaldehyde [92,93]. Self-healing, reprocessability, and shape memory properties were presented on the epoxy vitrimer [92], and similar abilities were confirmed for the imine-based CFRPs [93]. The degradation of the matrix material and the reclaim of the CFs were presented in both studies; however, the prepared systems had T_g_ values below ~100 °C.

Memon et al. [94] prepared a vanillin-based matrix with good mechanical properties and reprocessing ability after mechanical grinding and chemical degradation. The mechanical and chemical recycling of the matrix did not result in considerable deterioration of the thermomechanical response (E′ and T_g_ ~130 °C), while the study demonstrated the feasibility of reclaiming and reusing both the reinforcing phase (CF) and the matrix of the prepared CFRP. Another interesting element of the study was that it consolidated already cured laminas and compared the properties with the ones of conventionally manufactured CFRP vitrimers. A fully bio-based matrix was prepared in [95], starting from an epoxidized soybean oil and a curing agent from vanillin and 4-aminophenol. Although the study demonstrated the ability to reprocess the bio-based CFRP after grinding and compression molding, the mechanical performance of the recycled composite was inferior [95]. The hydrolysis of the matrix of the composite was also shown through its immersion in HCl solution with dimethylformamide (DMF) solvent and reclaiming almost intact CFs [95]. The authors continued their effort and combined the previously tested vanillin-based hardener with a commercial biobased resin to prepare CFRPs with good mechanical performance but relatively low T_g_ (approximately 70 °C) [96]. Apart from the ability to reprocess the vitrimer resin, without the deterioration of the thermo-mechanical properties, the dissolution of the matrix and reclaim of the CF was confirmed. The effect of curing agents on the performance of vanillin-based CFRPs was investigated by Wand et al. [97,98]. The authors prepared vitrimer matrices with higher T_g_ values (up to 102 and 132 °C, respectively) and confirmed the ability of the matrix to dissolve and provide clean and intact CFs. Furthermore, it was possible to use some of the matrix degradation products to reformulate vitrimer epoxies. Jiang et al. [99] prepared vanillin-based vitrimer CFRPs with quite high T_g_ (146 °C) and good mechanical properties. It was demonstrated that the matrix had the ability to be reprocessed with the application of heat and pressure and to degrade in acidic solvents. The prepared CFRPs were immersed in HCL solution and intact CFs were reclaimed.

The combination of bio-based imine-exchange chemistry with fire resistance characteristics in vitrimer CFRPs was investigated in [100,101,102,103,104,105,106]. The focus of the studies was more on the thermal, mechanical, and flame-retardant properties of the prepared epoxy vitrimers and respective CFRPs. The sustainability of the proposed materials was supported by their bio-based nature and ability to dissolve the matrix and reclaim the CF in acid-based solvents. Apart from the above, some of the studies have managed to propose matrix chemistries that result in very high T_g_ values (e.g., approximately 163 °C [101] and 234 °C [102]). Furthermore, Zamani et al. [106] proposed a synthesis route that involves the use of water as solvent and results in epoxy vitrimer with good mechanical performance and adequate T_g_ for various not-high-end applications (125 °C). Apart from dissolving the matrix to reclaim clean and high-performance CFs, the study presented the ability to separate the CFRP layers in HCl solution and remanufacture CFRPs via hot pressing with minor deterioration in stiffness and strength [106].

#### 3.2.3. Disulfide-Exchange Reactions

Thiol/disulfide exchange and disulfide exchange reactions have been widely discussed for the preparation of vitrimer epoxies since they can be activated at relatively low temperatures and require precursors that can be commercially available at reasonable costs [11,12,14]. However, disulfide bonds are relatively weak and may have durability issues due to the oxidation of free thiols and the deterioration of the stability of the S–S bond [12]. As observed in Table 4, which focuses on studies with disulfide bond exchange, there is a limited number of publications that employ such exchange reactions in epoxy-based CFRPs, while many overlaps are observed in the synthesis of the proposed matrix materials. In [107], starting from a commercial epoxy resin and introducing high contents of exchangeable aromatic disulfide crosslinks, it was possible to manufacture vitrimer CFRPs with mechanical and thermal performance that is adequate for structural applications (T_g_ of 147 °C for the resin) and reclaim/reuse the CF through the dissolution of the vitrimer matrix. The effect of the type and quantity of crosslinker on the mechanical and recycling performance of vitrimers based on diglycidyl ether of bisphenol A was investigated in [108]. As a proof of concept, a CFRP laminate was developed, and the matrix was dissolved to reclaim the CFs. The effect of the quantity of 4-aminophenyl disulfide was also investigated in [109]. The amount of the crosslinker was tuned to enable fast relaxation along with adequate mechanical and thermal performance, while the study demonstrated shape memory, shape-changing, and chemical recycling abilities for the CFRP [109]. Thermoformable and recyclable CFRPs were prepared in [110,111]. The mechanical recycling into short carbon fiber composites and thermoformability of pultruded CFRP profiles were validated in [110]. A thorough investigation of the thermoforming ability of vitrimer CFRPs was conducted in [111]. The focus of the study was on the level of defects that are induced in the structure after thermoforming, in direct comparison with the that observed in thermoplastic CFRP systems. The study concluded that thermoforming is challenging in vitrimer CFRPs due to the high viscosity of the matrix. The applied conditions need to be tuned to enable the thermoforming of systems with sulfur bonds with a small number of defects [111]. The only attempt to prepare bio-based epoxy vitrimer with S-S exchangeable bonds and manufacture respective CFRP composites is presented in [112]. The matrix had appreciable thermal/mechanical performance, while the feasibility of degrading the matrix of the CFRP, reusing the CFs, and reshaping the remaining matrix into films via hot-pressing was demonstrated [112].

#### 3.2.4. Other Exchange Reactions

Apart from the previous chemistries, the researchers have investigated other types of dynamic exchange reactions to develop vitrimer epoxies and respective CFRPs with enhanced performance, processability, and/or sustainability, which are included in Table 5. A few studies introduced acetal-based exchange reactions in the CFRP matrix [113,114,115] and presented the ability to reuse the CFs after the chemical degradation of the vitrimer resin. Systems with high toughness were prepared in [113], while the possibility of applying industrially relevant processing techniques was demonstrated in [114,115]. Apart from the ability of the chemical degradation of the matrix, the introduction of the ethyl group on the acetal ring resulted in low-viscosity vitrimers which enabled the application of vacuum-assisted resin transfer molding for the manufacturing of the CFRP [114]. Similarly, a low-viscosity, bio-based vitrimer with acetal bonds was prepared in [115] to assist in the resin transfer molding manufacturing of the CFRPs. The prepared systems showed very high T_g_ values (>226 °C) and the ability to degrade the matrix and reuse the CFs [115]. Other types of exchange reactions that have been explored for epoxy-based vitrimer CFRPs include dynamic benzyl ether [116], acylsemicarbazide [117], boronic ester [118], and diselenide [119] exchange bonds. It can be noted that in the case of the benzyl ether bond the chemical degradation of the vitrimer matrix was more complex and required the use of a catalyst [116]. The reaction between isocyanate and hydrazide compounds not only assisted the self-healing and the chemical recycling of the CFRP but enabled the reuse of the matrix degradation products, after upcycling, for the manufacturing of recycled CFRPs [117]. The boronic ester bonds can be easily degraded in ethanol [118], while the diselenide bonds were degraded in diselenide solution under UV light after 12 h [119].

Dual/multiple exchange bond reactions integrated within the vitrimer matrix and/or CFRP have been in the spotlight lately [120,121,122,123,124,125,126,127,128]. For instance, Ma et al. [120] introduced exchangeable bonds both on the surface of the CFs (thiol-functionalized) and within the epoxy resin (thiol-ended hyperbranched polymer) to develop high-performance composites with high-efficiency closed-loop recycling and managed to reuse both the matrix and the CFs to manufacture fully recycled CFRPs. Similarly, Li et al. [121] developed vitrimer CFRP with dynamic matrix (hyperbranched polyester + camphoric acid curing glycerol triglycidyl ether) and interface through the functionalization of the CFs. This direction was further explored by Zhang et al. [122]. The authors developed a hybridized structure with covalent bonds between a conventional epoxy, and an anhydride monomer, as well as supramolecular crosslinking via H-bonding and prepared systems with mild solvent degradation at RT and the reprocessability of the matrix, as well as extraordinary properties at low temperatures. Wang et al. [123] designed a triple crosslinked system with disulfide bonds and the silyl ether linkages. This resulted in CFRPs with shape memory ability, superior mechanical properties, and the recyclability of the CFs [123]. Xiang et al. [124] prepared CFRPs with good mechanical properties, stability, and solvent resistance through the introduction of dynamic disulfide and imine bonds in the vitrimer matrix, while the reclamation of the CFs was possible using two different solvent systems. In the same direction, a dual network with dynamic imine bonds and hydrogen bonds was presented in [125]. The proposed matrix demonstrated quite high toughness, and the ability to degrade in just 3 h due to the pH-sensitive feature of the network. Apart from reusing the reclaimed CFs, it was possible to use the decomposed matrix (monomers/oligomers) for toughening other thermosets [125]. The combination of acetal and disulfide bonds resulted in an epoxy vitrimer and respective CFRPs that can degrade in just 8 min in an acidic reducing solution [126]. Finally, bio-based dual dynamic networks were explored in [127,128]. Zhou et al. [127] prepared a bio-based vitrimer with a Schiff base and β-hydroxy ester dynamic bonds, while Verdugo et al. [128], incorporated imine and disulfide bond exchanges in the bio-based epoxy. Both studies conclude that the introduction of two types of exchange reactions can result in the versatility and tuning of the hardeners/conditions applied for the activation of the bond exchange reactions.

#### 3.2.5. Comparison of the Different Vitrimer-Based CFRPs

**Figure 8:**
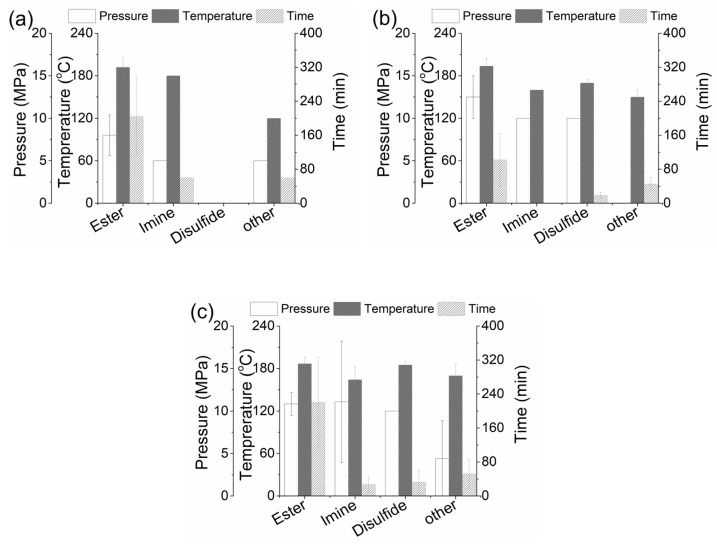
Comparison of the processing conditions applied for: (**a**) healing/repairing, (**b**) welding/reshaping, and (**c**) mechanical recycling of vitrimer-based CFRPs.

Based on the information presented in Table 2, Table 3, Table 4 and Table 5, a direct comparison of the different processing conditions used for the life extension and/or end-of-life management of the vitrimer-based CFRPs is attempted. Figure 8 compares the pressure, temperature, and time required for the delamination healing/repairing (Figure 8a), welding/reshaping (Figure 8b), and mechanical recycling, i.e., reprocessing (Figure 8c), depending on the type of vitrimer chemistry applied for the manufacturing of the dynamic CFRPs. As observed in Table 2, Table 3, Table 4 and Table 5 and Figure 8 the available information is not complete, especially around the applied processing conditions of vitrimer CFRPs with imine-, disulfide-, and other exchange reactions. However, based on the available data, it can be deduced that for the healing/re-pairing of delaminations in CFRPs, ester-based exchange reactions are the most demanding followed by imine-exchange ones (Figure 8a). Looking into the processing conditions used for the welding and/or reshaping processes, the ester-based reactions require slightly higher temperatures compared to the other chemistries (Figure 8b). Furthermore, imine- and disulfide-chemistries require similar conditions in terms of pressure and temperature for stress relaxation/reshaping. Dual/multiple exchange bond reactions favor the reprocessing of chopped CFRPs at lower pressure during mechanical recycling, while imine- and disulfide-bond exchange reactions are the quickest ones (Figure 8c). It should also be noted that considerable variation is observed in the processing conditions, even within the same matrix category. Since all processes presented in Figure 8 depend on the rate of topological changes, that translates into stress relaxation, the rate of the bond exchange of the different chemistries, and the activation energy of the bond exchange dictates the processing conditions. As already mentioned, these observations require further validation from more data, especially for imine-, disulfide-, and other dynamic systems, where limited information is available.

One of the biggest challenges of conventional thermoset composites over their counterparts with a thermoplastic matrix is related to their inability to thermoform. Thermoforming is a manufacturing technique with high production rates, that could also be utilized for reshaping end-of-life composites. While most of the research articles reviewed in § 3.2 try to confirm the feasibility of reshaping/reforming the CFRPs through the introduction of exchange reactions, in most cases, the research is restricted to simple hot-pressing experiments on the vitrimer matrix and/or CFRP composites under different processing conditions that vary in terms of time, temperature, and pressure, as observed in Figure 8b. Only one attempt [111] has been made to thoroughly investigate the thermoforming ability of vitrimer CFRPs, and the main conclusion was that vitrimer matrices with low viscosities are required to support defect-free thermoforming [111]. Mechanical recycling of vitrimer CFRPs is based on the relaxation of the vitrimer matrix and its respective flow upon the application of heat and pressure. While the same mechanism prevails also under welding/reshaping, it can be observed (Figure 8c) that slightly higher temperatures are applied for the mechanical recycling of vitrimer CFRPs.

**Figure 9:**
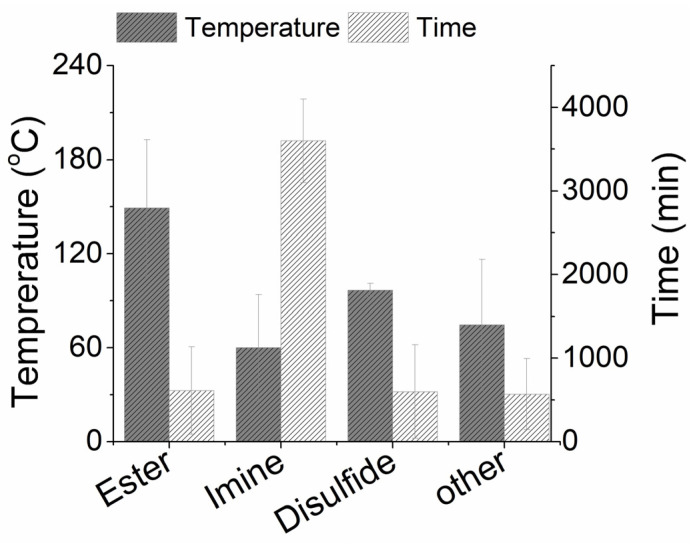
Comparison of the processing conditions applied for chemical recycling of vitrimer-based CFRPs.

The average processing conditions for the chemical recycling of the different vitrimer-based CFRPs are illustrated in Figure 9. The average processing time is similar among CFRPs with ester-, disulfide-, and other dynamic exchange bonds. The respective time is considerably higher in the case of dynamic composites with imine-exchange bonds, which is, however, counterbalanced by the considerably lower average temperature selected for the dissolution of the dynamic matrix for such systems. In terms of the toxicity of the involved solvents, in the reviewed studies, ester-based CFRPs are dissolved using water, and/or class 2 (e.g., EG, TFH, etc.) and class 3 (e.g., ethanol/acetone, etc.). Imine-based systems mainly use class 2 solvents (e.g., HCl/THF, DMF), and the same holds for disulfide-based CFRPs (e.g., DMF). Depending on the exchange chemistry, the solvents used in other dynamic CFRPs range from water to class 2 solvents.

## 4. Conclusions—Outlook

The current review paper attempts to discuss the recent efforts of the research community to create CFRP composites with epoxy vitrimers, and the reflection of such efforts on the life extension and end-of-life management options of the CFRP composites. Based on this analysis, the following conclusions can be drawn:

- The beneficial characteristics of vitrimer matrices over repairability were mostly demonstrated in the example of surficial cracks applied on the matrix.
- Reshaping/reprocessing was mainly examined under “lab-scale” demo cases.
- A proof-of-concept of reprocessing/mechanical recycling via grinding of the matrix material and remolding through hot-pressing has been presented with limited information on the reprocessing of chopped vitrimer-based CFRPs [110].
- A demonstration of the feasibility of the chemical decomposition of the vitrimer matrix of the CFRPs and the reclaim of intact CFs have been provided.
- The possibility of decomposing the vitrimer matrix at RT was also demonstrated; however, stronger and potentially more toxic solvents were required.

Based on these conclusions, some suggestions for future works are the following:

- To fully demonstrate the possibility of repairing vitrimer-matrix composites, it is imperative to conduct studies that simulate better the failure of CFRPs under different types of loadings. On-site repair options would also be beneficial for the large-scale application of vitrimer CFRPs.
- To make CFRPs with dynamic bonds competitive in terms of reshaping/reprocessing, it would be beneficial to prove their thermoforming ability under the conditions conventionally used to process thermoplastic-based CFRPs.
- More research that would relate the processing conditions, along with various aspects of the formed part (e.g., geometry, fiber layup, thickness distribution, etc.), with the level of defects that are induced in the structure after thermoforming, is required [129,130].
- For the future application of the vitrimer CFRPs after mechanical recycling, it would be beneficial to define the span of the obtained properties of the short-fiber reinforced composites as a function of the grinding/reprocessing conditions.
- The reclaim and reuse of the matrix decomposition products after chemical recycling as well as their upcycling requires further investigation since this is an understudied but important field.

Overall, it can be concluded that vitrimer composites offer distinct benefits over conventional CFRPs in terms of life extension possibilities; however, in terms of recycling, their sustainability profile, especially in terms of chemical recycling, needs to be directly compared with that of conventional CFRP composites through life-cycle analysis studies.

## Figures and Tables

**Table 1:** Indicative recent publications on chemical recycling of CFRPs with conventional epoxy matrix.

Conditions (e.g., Solvent Type, Temperature, Pressure, Duration)	Ref.
Pretreatment of CFRP with glacial acetic acid at 90 °C for 40 min and decomposition in monoethanolamine (MEA) solvent with potassium hydroxide at 160 °C for 90 min—93% of the mechanical properties of virgin CFs	[57]
Pretreatment of CFRP with citric acid solution at 120 °C for 6 h and decomposition in meta-chloroferoxybenzoic acid + dichloromethane at 40 °C for 6 h—93% of the tensile strength of virgin CFs	[56]
Decomposition of CFRP (after swelling in N-methyl-2-pyrrolidone) in 30 % or 50% H_2_O_2_ solution at 106–115 °C and 175–195 °C for 8–39 h—almost the same single fiber tensile strength and Young’s modulus as of virgin CFs	[58]
Swelling of the CFRP in acetic acid at 100 °C, 120–220 °C, 0.07–1.03 MPa, for 0.5–4 h—the CFRP was either reclaimed in a softer form or in a delaminated one and with the addition of fresh epoxy reprocessed in new CFRPs with 47–89% of flexural strength compared with the original CFRP	[59]
Mixture of deep eutectic solvents (DESs) and metal salt catalysts (e.g., ZnCl_2_, FeCl_2_, and FeCl_3_)—Complete epoxy resin matrix decomposition under ordinary pressure and mild temperature (180 °C for 2 h)—94.5% of the original strength was retained	[60]
Decomposition of CFRP in Al(NO_3_)_3_·9H_2_O (catalyst) and dimethylacetamide (solvent) at 160 °C for 5 h—comparable mechanical and chemical properties of recycled CFs, possibility to reuse the recovered epoxy matrix, possibility to reuse the initial solvent	[61]
98.82 % decomposition of CFRP in monoethanolamine solution with potassium hydroxide at 160 °C for 90 min—93% retention of the original tensile strength	[57]

**Table 2:** Overview of publications on vitrimer CFRPs with ester-exchange reactions—information on the healing/repairing, welding/reshaping, and/or recycling results of the studies.

Chemistry of Vitrimer Matrix in CFRPs	Healing/Repairing	Welding/ Reshaping	Recycling (Mechanical/ Chemical)	Ref.
bisphenol A diglycidyl ether + ethylenediamine + glutaric anhydride	-	shape-changing of the CFRP at 200 °C for 1 h	hydrothermal degradation of the vitrimer matrix of the CFRP at 180 °C for 5 h—reclaim of the CF	[70]
bisphenol A + bisphenol F diglycidyl ether + glutaric anhydride + Zn-based catalyst	delamination healing of CFRPs via molding (10 MPa, 200 °C, 4 h)	rebonding of cut matrix specimens via molding (10 MPa, 180–200 °C, 4 h)	reprocessing of chopped epoxy vitrimer via hot pressing (200 °C, 10 MPa pressure, 4 h)	[71]
bisphenol A + bisphenol F diglycidyl ether + glutaric anhydride + Zn-based catalyst + PLA-based microchannels	delamination healing of CFRPs (8 MPa, 180 °C, 2 h) and healing agents through external channels	-	-	[72]
bisphenol A glycidyl ether + glutaric anhydride + carboxy-terminated polyether + Zn-based catalyst	crack healing of epoxy vitrimer (150 °C, 5 min)	welding of epoxy vitrimer (150 °C, 3 h), deformation and recovery of epoxy vitrimer (90, 180 °C)	reprocessing of chopped epoxy vitrimer via hot pressing (180 °C, 10 MPa, 6 h), chemical degradation of the vitrimer matrix of the CFRPs in EG solution (180 °C, 6 h)—reclaim of CF	[73]
poly[(phenylglycidyl ether)-coformaldehyde + dicarboxylic acid + Zn-based catalyst	-	crack healing/Welding CFRP (180 °C, 30 min)	chemical degradation of the vitrimer matrix of the CFRPs in ethanol/acetone solution (50:50) (180 °C, 4 h, pressurized reactor)	[74]
bisphenol A diglycidyl ether + tetrahydro-methyl phthalic anhydride + 2,4,6-tris (dimethyl aminomethyl) phenol + Zn-based catalyst	reparability of delamination in CFRPs with hot-pressing (20 bar, 220 °C, 1 h)	rebonding of CFPRs via using of vitrimer matrix as adhesive (200 °C, 1 h) and thermoforming of CFRPs	-	[75,76]
diglycidyl 4,5-epoxycyclohexane-1,2-dicarboxylate + phthalic anhydride + glycerol	self-healing data only for the epoxy vitrimer (230 °C, 2 h)	shape-changing and shape-memory performance for the epoxy vitrimer (230 °C, 2 h)	chemical degradation of the vitrimer matrix of the CFRPs at 190 °C after 11 h by using ethylene glycol (EG)—reclaim of the CF	[77]
tetraglycidyl methylenedianiline epoxy + nadic methyl anhydride + triethanolamine	-	-	hydrothermal degradation of the vitrimer matrix of the CFRP at 200 °C for 5 h—reclaim of CF	[78]
resin monomer + glutaric anhydride + Zn-based catalyst	-	-	degradation of the vitrimer matrix of the CFRP in EG at 160 °C (app. 5–15 h) after thermo-oxidative aging	[79]
bisphenol A diglycidyl ether + sebacic acid + 1,5,7-triazabicyclo[4.4.0]dec-5-ene	repair of composite specimens after ILSS testing with hot pressing	-	-	[80]
bisphenol A diglycidyl ether + adipic acid + phosphaphenanthrene-based diol	-	-	chemical degradation of the vitrimer matrix of the CFRPs at 180 °C after 5 h by using ethylene glycol (EG)—reclaim of the CF	[81]
bisphenol A diglycidyl ether + phosphorus-containing diol with tertiary amine structure + methylhexahydrophthalic anhydride	healing of CFRPs with hot pressing (180 °C, 10 MPa, 4 h)	-	reprocessing of chopped epoxy vitrimer via hot pressing (180 °C, 10 MPa pressure, for different times), chemical degradation of the vitrimer matrix of the CFRP in EG solution at 180 °C for 10 h—reclaim of the CF	[82]
bisphenol A diglycidyl ether + phosphaphenanthrene-derived anhydride + triethanolamine	healing of CFRPs with hot pressing (180 °C, 10 MPa, 6 h)		reprocessing of chopped epoxy vitrimer via hot pressing (180 °C, 10 MPa pressure, for different times), chemical degradation of the vitrimer matrix of the CFRP at 160 °C after 3 h in ethanolamine—reclaim of the CF	[83]
tetraglycidyl methylene diphenylamine + phosphorus-containing anhydride + triethanolamine	-	shape memory of the vitrimer matrix	hydrothermal of the vitrimer matrix of the CFRP at 200 °C after 5 h—reclaim of the CF and matrix degradation products	[84]
bisphenol A diglycidyl ether + tertiary amine/phosphorus-containing reactive oligomer + Methyl tetrahydrophthalic anhydride	-	-	chemical degradation of the vitrimer matrix of the CFRPs in tetrahydrofuran (THF)/NaOH (volume ratio = 8:2) at 50 °C after 72 h—reclaim of the CF and matrix degradation products	[85]
bio-based epoxidized menthane diamine + adipic acid	self-healing data only for the epoxy vitrimer (180 °C for 10–60 min)	reshaping, shape memory, and welding ability (100–180 °C) and pressure for different times	reprocessing of chopped epoxy vitrimer via hot pressing (180 °C, 1 h, 15 MPa pressure), chemical degradation of the vitrimer matrix of the CFRPs at 100 °C after 30 min in ethanolamine—reclaim of the CF	[86]
epoxidized soybean oil + natural camphoric acid + 1,5,7-triazabicyclo[4.4.0]dec-5-ene	reduction in crack and healing (200 °C, 10 min, EG)	reshaping, shape memory, and welding ability (100–200 °C) for different times	degradation of the vitrimer matrix of the CFRP in EG at 190 °C for 20 h—reclaim of the CF	[87]
bisphenol A diglycidyl ether + Tung oil-based acid/anhydride + tris(dimethyl-amino-methyl)phenol + Zn-based catalyst	reduction and healing of a surficial crack on the epoxy vitrimer (180 °C, 5–60 min)	shape-changing and shape-memory performance for the epoxy vitrimer	reprocessing of chopped epoxy vitrimer, chemical degradation of the vitrimer matrix of the CFRP with ethanol and NaOH after 12 h of microwave heating at 100 °C—and reclaim of CF	[88]
epoxy monomers + Tung oil-based acid curing agents	reduction and healing of a surficial crack on the epoxy vitrimer (180 °C, 60 min)	shape-changing and shape-memory performance only for the epoxy vitrimer	reprocessing of chopped epoxy vitrimer via hot pressing, chemical degradation of the vitrimer matrix of the CFRPs at 100 °C after 60 min by using ethanolamine	[89]
epoxy dicardanol-based succinate/epoxy dicardanol-based adipate + hexahydro-4-methylphthalic anhydride + 1, 5, 7-triazabicyclo[4.4.0]dec-5-ene	self-healing data only for the epoxy vitrimer (150 °C, 30–180 min)	shape-changing (180 °C, 30 min), and welding ability of the CFRPs with the application of 150 °C and 15 MPa	reprocessing of chopped epoxy vitrimer via hot pressing (10 MPa at 200 °C), chemical degradation of the vitrimer matrix of the CFRPs in NaOH–ethanol at RT for 2 h– reclaim of the CF	[90]
rosin derived glycidyl ester epoxy resin + vegetable oil derived sebacic acid	reduction and healing of a surficial crack on the epoxy vitrimer (180 °C, 30 min)	shape-memory performance by heating above the T_g_—cooling to RT	hydrothermal degradation of the vitrimer matrix of the CFRP at 160 °C for 2 h—reclaim of the CF	[91]

**Table 3:** Overview of publications on vitrimer CFRPs with imine-exchange reactions—information on the healing/repairing, welding/reshaping, and/or recycling results of the studies.

Chemistry of Vitrimer Matrix in CFRPs	Healing/Repairing	Welding/ Reshaping	Recycling (Mechanical/ Chemical)	Ref.
bisphenol A epoxy resin + Jeffamine D-230 polyetheramine + terephthalaldehyde	self-healing data only for the epoxy vitrimer (130–150 °C, 8–30 min)	reshaping, shape memory, and welding ability for the epoxy vitrimer	reprocessing of chopped epoxy vitrimer via hot pressing (150 °C, 12 h), chemical degradation of the vitrimer matrix of the CFRPs in HCl solution (solvent water/THF), RT, 24 h—reclaim of CFs	[92]
bisphenol A epoxy resin + Jeffamine EDR-148 polyetheramine + terephthalaldehyde	-	shape-changing (180 °C, 1 h), shape-memory, and welding ability of the CFRPs with the application of 180 °C and 1 MPa	chemical degradation of the vitrimer matrix of the CFRPs in HCl solution at 200 °C for 4 h—reclaim of CFs	[93]
trifunctional epoxy resin + Ethylene glycol diglycidyl ether + imine-containing hardener based on vanillin and methylcyclohexanediamine	reparability of CFRPs via hot pressing (180 °C, 5 MPa, 1 h)	-	reprocessing (up to 3 times) of chopped epoxy vitrimer via hot pressing (180 °C, 5 MPa, 40 min), chemical degradation of the vitrimer matrix of the CFRPs in ethylenediamine (100 °C, 2 h)—reclaim and reuse of CFs and epoxy matrix	[94]
epoxidized soybean oil + curing agent from vanillin and 4- aminophenol + 1, 2-dimethylimidazole	-	-	reprocessing of chopped CFRP via hot pressing (180 °C, 20 MPa, 10 min), Acid hydrolysis of the vitrimer matrix of the CFRPs in HCl solution (solvent: DMF) (RT, 20 h)—reclaim of CFs	[95]
glycerol triglycidyl ether + curing agent from vanillin + 4-aminophenol + 1, 2-dimethylimidazole	-	-	reprocessing of chopped epoxy vitrimer via hot pressing (140 °C, 10 min, 20 MPa), chemical degradation of the vitrimer matrix of the CFRPs in ethylenediamine solution (DMF solvent) (50 °C, 2 h)—reclaim of CFs	[96]
vanillin-based epoxy monomer with aldehyde group + 4,4′-diaminodiphenylmethane or diethylenetriamine or isophoronediamine or polyetheramine	-	-	chemical degradation of the vitrimer matrix of the CFRPs in HCl solution (RT, 24 h) or HCl/DMF solution (RT, 2 h)—reclaim of CF and in some cases matrix degradation products	[97,98]
vanillin + 4-aminocyclohexanol + epichlorohydrin + tetrabutylammonium bromide + 4,40-diaminodiphenylmethane	-	-	reprocessing of chopped epoxy vitrimer via hot pressing (180 °C, 0.5 h), chemical degradation of the vitrimer matrix of the CFRPs in HCl solution —reclaim of CFs	[99]
two vanillin based epoxy vitrimers + 9,10-dihydro-9-oxo-10-phosphaphenanthrene-10-oxide) + diamino diphenylmethane)	repairability of epoxy vitrimer (60 °C, 24 h)	reprocessing of epoxy vitrimer hot pressed by a plate vulcanizer at 140 °C and 10 MPa	reprocessing of chopped epoxy vitrimer via hot pressing (140 °C, 10 MPa, 10 min) chemical degradation of the vitrimer matrix of the CFRPs in HCl/THF solution at 60 °C for 4 h—reclaim of CFs	[100]
bio-based epoxy monomer from vanillin and tyramine + bio-based curing agent from furfurylamine	-	-	chemical degradation of the vitrimer matrix of the CFRPs in HCl/THF solution (50 °C)—reclaim of CFs	[101]
furan-derived epoxy + furan-based curing agent	-	-	chemical degradation of the vitrimer matrix of the CFRPs in HCl/THF solution at RT for 24 h—reclaim of CFs	[102]
bisphenol A diglycidyl ether + vanillin derived dynamic crosslinker + 9,10-Dihydro-9-oxa-10-phosphaphenanthrene-10-oxide + 5-amino-1H-tetrazole + p-hydroxybenzaldehyde	-	-	chemical degradation of the vitrimer matrix of the CFRPs in ethylenediamine solution at 80 °C for 3 h—reclaim of CFs	[103]
bisphenol A diglycidyl ether + vanillin derived dynamic crosslinker + 10-(2,5-dihydroxyphenyl)-10-hydro-9-oxa-10-phosphaphenanthrene-10-oxide	-	-	chemical degradation of the vitrimer matrix of the CFRPs in ethylenediamine solution at 80 °C for 4 h—reclaim of CFs	[104]
bisphenol A diglycidyl ether + vanillin derived dynamic crosslinker	-	shaping of CFRP at controlled force (160 °C)	chemical degradation of the vitrimer matrix of the CFRPs in HCl/THF solution at RT for 5 h—reclaim of CFs	[105]
vanillin-based monomer + hexamethylenediamine		-	chemical degradation of the vitrimer matrix of the CFRPs in HCl/THF, at 60 °C for 5 h—reclaim of CFs, delamination and separation of CFRP layers in HCl solution, at 100 °C—remanufacturing of CFRPs after hot pressing at 180 °C under 4 bar pressure for 1 h	[106]

**Table 4:** Overview of publications on vitrimer CFRPs with disulfide-exchange reactions—information on the healing/repairing, welding/reshaping, and/or recycling results of the studies.

Chemistry of Vitrimer Matrix in CFRPs	Healing/Repairing	Welding/ Reshaping	Recycling (Mechanical/ Chemical)	Ref.
bisphenol A diglycidyl ether + bis(4-glycidyloxyphenyl)disulfide/4-aminophenyl disulfide	-	-	reprocessing of chopped epoxy vitrimer via hot pressing (1 h at 180 °C), chemical degradation of the vitrimer matrix of the CFRPs in dithiothreitol (DTT)/DMF, at 90 °C for 1 h—reclaim of CFs and reuse to manufacture CFRPs	[107]
bisphenol A diglycidyl ether + 4-aminophenyl disulfide or 2- aminophenyl disulfide	-	-	chemical degradation of the vitrimer matrix of the CFRPs in DMF/2- mercaptoethanol(2ME), at 100 °C for 24 h—reclaim of CFs	[108]
glycerol triglycidyl ether + 4-aminophenyl disulfide	self-healing of the vitrimer matrix	reshaping of CFRP (180 °C, 30 min), shape memory (120 °C)	chemical degradation of the vitrimer matrix of the CFRPs in DTT/DMF solution, at 100 °C for 5 h—reclaim of CFs	[109]
bisphenol A diglycidyl ether + 4-aminophenyl disulfide	-	thermoforming of pultruded CFRP (190 °C, 100 bar for 10 min)	reprocessing of chopped CFRP via hot pressing at 190 °C, 100 bar for 5 min	[110]
mixture of bisphenol F diglycidyl ether/tetraglycidyl-4,4′ methylene dianiline + 4-aminophenyl disulfide	-	thermoforming via heating (3-point bending or hot press, various temperatures)	-	[111]
guaiacol-based epoxy resin + 4-aminophenyl disulfide	-	remolding of the epoxy vitrimer (180 °C, 10 MPa for 15 min)	chemical degradation of the vitrimer matrix of the CFRPs in DMF/β-ME (50/50) at RT for up to 7 days—reclaim of CFs and reprocess of the matrix via hot-pressing	[112]

**Table 5:** Overview of publications on vitrimer CFRPs with other types of exchange reactions (e.g., acetal, dual, etc.)—information on the healing/repairing, welding/reshaping, and/or recycling results of the studies.

Chemistry of Vitrimer Matrix in CFRPs	Healing/Repairing	Welding/ Reshaping	Recycling (Mechanical/ Chemical)	Ref.
2,2-bis(4-hydroxycyclohexyl)propane or 2,2-bis[4-(2-hydroxyethoxy)phenyl]propane (HOBA) + cyclohexane dimethanol vinyl glycidyl ether	-	-	hydrolysis of the vitrimer matrix of the CFRPs under acidic conditions—reclaim of CFs	[113]
acetal diols + tetrabutylamine bromide + epichlorohydrin + isophorone diamine	-	-	chemical degradation of the vitrimer matrix of the CFRPs in HCl acetone/water solution at RT for 24 h—reclaim of CFs and reuse to manufacture CFRPs	[114]
bio-based diglycidyl ether pentaerythritol vanillin diacetal + 4,40-diaminodiphenyl sulfone + low viscosity bisphenol A	-	-	chemical degradation of the vitrimer matrix of the CFRPs in hydrochloric acid/THF/water mixed solvent at RT for 6 h—reclaim of CFs	[115]
1,4-Bis((2-oxiranylmethoxy)methyl)- Benzene + 4,4′-diamino diphenyl sulfone (for the CFRPs)	-	-	chemical degradation of the vitrimer matrix of the CFRPs in benzyl methanol with AlCl_3_ as catalyst (190 °C, 3 h)—reclaim of CFs	[116]
sebacic dihydrazide + isophorone diisocyanate + hexamethylene diisocyanate trimer + *N*,*N*-dimethylformamide	self-healing of delamination damage under hot pressing (120 °C, 5 MPa, 1 h)	-	chemical degradation of the vitrimer matrix of the CFRPs in DMF at 120 °C for 2 h—reclaim of CFs and the matrix via volatilizing the solvent in an oven—use CFs and upcycled matrix for the preparation of CFRPs	[117]
epoxidized linseed oil + diboronic ester dithiol dynamic cross-linker	-	shape memory of the vitrimer matrix	compression molding of cut matrix specimens for 10 min at 160 °C using a load of 5 tons—chemical degradation of the vitrimer matrix of the CFRPs in ethanol at 75 °C for 3 h—reclaim of CFs	[118]
epoxy resin + diselenide-labeled diaromatic amine 4,4′ -diselanediylbis(4-amino-N-benzylbutanamide)	repairing of the epoxy vitrimer via hot-pressing (0.1 bar, 170 °C)	reshaping ability via hot-pressing	reprocessing of chopped epoxy vitrimer via hot pressing (10 bar at 170 °C for 10 min), chemical degradation of the vitrimer matrix of the CFRPs in diselenide solution under UV at 40 °C for 12 h—reclaim of CFs	[119]
epoxy-ended hyperbranched polymers containing degradable hexahydro-s-triazine + thiol-ended hyperbranched polymer coated CFs	-	-	CFRPs were degraded selectively into monomers, oligomers, CFs H_3_PO_4_/THF or HCOOH/THF solutions—all materials were reused to manufacture vitrimer resin (after neutralization and addition of formaldehyde) and CFRPs	[120]
hyperbranched polyester + camphoric acid curing glycerol triglycidyl ether + functionalized CFs by polydopamine	self-healing of cracks/holes in CFRPs (120 °C, 30 min) and interfacial failures	-	reprocessing of chopped epoxy vitrimer via hot pressing (10 MPa at 200 °C for 2 h), chemical degradation of the vitrimer matrix of the CFRPs in ethylene glycol at 180 °C after 8 h—reclaim of CFs	[121]
bisphenol A diglycidyl ether + methyl tetrahydrophthalic anhydride + hyperbranched phosphate/borate hybrid polymer	-	-	degradation was examined only for the matrix material	[122]
diglycidyl ether of bisphenol F + 4-aminophenyl disulfide + γ-aminopropyltriethoxysilane + poly(propylene glycol) bis(2-aminopropyl ether)	-	triple-shape memory performance of the CFRPs (130 °C, 70 °C, RT)	chemical degradation of the vitrimer matrix of the CFRPs in DMF/ME solution (24 h)	[123]
bisphenol A diglycidyl ether + amino phenyl diimine + aminophenyl disulfide	-	-	chemical degradation of the vitrimer matrix of the CFRPs in DTT/DMF solutions at 80 °C for 8 h, and then in HCl/THF solutions at 50 °C for 5 h	[124]
bisphenol A diglycidyl ether + 4-Imidazole formaldehyde + polyether amine	-	-	chemical degradation of the vitrimer matrix of the CFRPs in HCl/THF solution at 50 °C for 24 h—reclaim of CFs, reclaim of the matrix degradation products	[125]
*p*-hydroxybenzaldehyde + di- (trimethylolpropane) + epichlorohydrin + cystamine	healing of a surface crack (180 °C, 60 min)	-	reprocessing of chopped epoxy vitrimer via hot pressing (180 °C, 10 MPa for 1 h), chemical degradation of the vitrimer matrix of the CFRPs in a mixture of THF/*β*-He/H_2_O, HCl solution at 25 °C—reclaim of CFs	[126]
rosin-based epoxy resin + benzoxazine + 1,8-menthane diamine + acrylpimaric acid diglycidyl ester	-	self-adhesion of the CFRPs after hot-pressing at 160 °C, 1.5 MPa, for 30 min, reshaping (160 °C, 10 min), shape-memory (100 °C, 30 min)	chemical degradation of the vitrimer matrix of the CFRPs in n-butylamine (60 °C for 30 min)	[127]
cystamine bis(vanillin glycidyl ether) + 5-amino-1,3,3-trimethylcyclohexanemethylamine		self-adhesion and reshaping of CFRPs (140 °C, 1 h, under pressure)	reprocessing of chopped epoxy vitrimer via hot pressing (140 °C, 0.4 MPa for 1 h), chemical degradation of the vitrimer matrix of the CFRPs and reclaim of CFs in a) HCl solution of H_2_O:THF at RT for 24 h, and b) DTT solution in DMF at 50 °C for 4 h	[128]

## Data Availability

No new data were created or analyzed in this study. Data sharing is not applicable to this article.
